# The efficacy and safety of targeted narrowband UVB therapy: a retrospective cohort study

**DOI:** 10.3906/sag-1810-110

**Published:** 2019-04-18

**Authors:** Kübra ESEN SALMAN, İlknur KIVANÇ ALTUNAY, Andaç SALMAN

**Affiliations:** 1 Department of Dermatology, Tuzla State Hospital, İstanbul Turkey; 2 Department of Dermatology, Şişli Hamidiye Etfal Training and Research Hospital, İstanbul Turkey; 3 Department of Dermatology, School of Medicine, Marmara University, İstanbul Turkey

**Keywords:** Alopecia areata, lichen simplex chronicus, psoriasis, targeted phototherapy, ultraviolet B, vitiligo

## Abstract

**Background/aim:**

Phototherapy is a safe and effective treatment modality for numerous dermatological conditions. Recently, targeted phototherapy modalities have gained importance due to their advantages over conventional phototherapy. This retrospective study aimed to evaluate the safety and efficacy of targeted narrowband UVB phototherapy in patients with dermatological disorders.

**Materials and methods:**

This single-center study included 173 patients who were treated with targeted narrowband UVB phototherapy. Demographic features, phototherapy parameters, and adverse effects were evaluated in all patients, and the treatment response was assessed in patients who attended at least one follow-up visit.

**Results:**

A total of 173 patients (102 females; 71 males) with vitiligo, alopecia areata, lichen simplex chronicus, palmoplantar psoriasis, and psoriasis vulgaris were included in the study. Among 73 patients, with whom the treatment was finalized by physician, an excellent response was obtained in 10%, 52.9%, 53.8%, 28.6%, and 40% of patients with vitiligo, alopecia areata, lichen simplex chronicus, palmoplantar psoriasis, and psoriasis, respectively. The treatment was generally well tolerated and was discontinued in only two patients due to adverse effects.

**Conclusion:**

This study demonstrates that targeted narrowband UVB therapy is a safe and effective treatment alternative, particularly for alopecia areata, lichen simplex chronicus, and palmoplantar and plaque-type psoriasis.

## 1. Introduction

Phototherapy is a safe and effective treatment method that utilizes ultraviolet radiation (UVR). Phototherapy has an immunosuppressive effect on cutaneous T cells and cytokines, and it is used in the treatment of various dermatological conditions, including psoriasis, cutaneous T cell lymphoma, and vitiligo (1). However, conventional phototherapy methods have certain limitations in localized diseases due to their acute and chronic adverse effects. To that end, microphototherapy or targeted phototherapy technologies have been developed. Targeted phototherapy has several advantages over conventional methods, such as not exposing healthy skin areas to UVR and shorter treatment durations; as a result, the inconvenience experienced by the patient lessens, and the patient satisfaction and adherence to treatment increases (2–6).

Several studies have investigated the efficacy of microphototherapy, particularly for vitiligo and psoriasis (3–5). However, the safety and efficacy of targeted phototherapy in other dermatological diseases have not been studied in detail.

The present study aimed to retrospectively evaluate the safety and efficacy of NB-UVB microphototherapy in various dermatological disorders.

## 2. Materials and methods

A retrospective chart review study was planned in order to evaluate the safety and efficacy of targeted UVB therapy in various cutaneous diseases. Our institutional ethics committee reviewed and approved the study (approval date/number: 09.12.2014/803). 

### 2.1. Patient selection

All patients referred to our phototherapy unit for targeted UVB therapy between 2014 and 2016 were included in the study. All patients were unresponsive to previous topical and/or systemic treatment attempts except for 18 patients (10.5%) who did not receive any treatment before. The medical charts and clinical photographs of all patients were reviewed in order to analyze patient demographics, disease and treatment characteristics, phototherapy parameters, treatment outcomes, and adverse effects. 

### 2.2. Treatment protocol and its implementation

In our phototherapy unit, targeted UVB therapy was performed with a Daavlin-Levia device (Bryan, Ohio, USA) emitting NB-UVB radiation with a wavelength of 311–315 nm; the device is capable of treating an area of 3 cm2 with an output of 90 mW/cm2.

The minimal erythema dose (MED) was calculated for the back skin in all patients prior to treatment. 

The treatment was applied two or three times a week, and the initial dose was determined as 30% of the MED for patients with vitiligo and 50% of the MED for patients with other conditions. The dose increments were conducted at every session by evaluating the erythema response, usually by 50 mJ/cm2 for patients with vitiligo and 20% of the last dose for patients with other conditions. The adverse effects associated with phototherapy, such as erythema, pigmentation, itching, and bullae formation, were evaluated before every session.

Clinical photographs of the patients were taken prior to treatment and then every 4 weeks with the same camera, in the same room, and with the same light conditions. The treatment was continued until complete clearance or maximum efficacy was achieved and discontinued when patients had worsening lesions or side effects.

### 2.3. Subjective evaluation of severity of disease

Patients with lichen simplex chronicus (LSC), palmoplantar psoriasis/eczema, and psoriasis vulgaris were asked to subjectively evaluate the severity of pruritus caused by their dermatological disorder every 4–8 weeks using a visual analog scale (VAS) (0–10 cm).

### 2.4. Evaluation of response to treatment

The same dermatologist (KES) evaluated the response to treatment by comparing the photographs and using the physician’s global assessment.

In patients with alopecia areata (AA), the response to treatment was assessed according to the rate of terminal and vellus hair regrowth. In patients with vitiligo, the response to treatment was evaluated according to the rate of repigmentation; in patients with psoriasis, the presence and improvement rate of erythema, desquamation, and infiltration; in patients with LSC, the severity of lichenification; and in patients with palmoplantar psoriasis/eczema, the presence and improvement rate of infiltration, erythema, desquamation, and fissures were compared between pre- and posttreatment photos. In patients with more than one lesion treated, final response to treatment was determined through the global assessment of all treated lesions. Accordingly, the treatment response was defined as follows: no response (0%–25%), mild response (25%–50%), moderate response (51%–75%), significant response (76%–90%), and complete response (>90%).

### 2.5. Statistical analysis

Statistical analysis was performed using SPSS 21.0 (IBM Corp., Armonk, NY, USA). Descriptive statistical methods were used in the analysis of the data. The mean, standard deviation, and range were calculated in the analysis of numerical variables. Categorical variables were assessed by frequency analysis.

Although the occurrence of side effects and phototherapy parameters were analyzed in all patients who had at least one treatment session, the response to treatment was only assessed in patients who had attended at least one follow-up visit during the treatment course. The last observation carried forward was used for the analysis of treatment outcomes in those patients who did not attend a follow-up. The response rates were also evaluated separately in patients whose treatment was finalized by a physician. 

## 3. Results

A total of 173 patients were referred to our phototherapy unit during the study period, whose demographic and disease characteristics are summarized in Table 1. Sixteen (9.2%) of the 173 patients were excluded from the study as a result of nonattendance in the follow-up process. The remaining 157 (91.8%) patients received more than one session of therapy and attended at least one follow-up visit.

**Table 1:** Demographic and disease characteristics of the patients.

Characteristics		Vitiligo	Alopecia areata	Lichen simplex chronicus	Palmoplantar psoriasis/eczema	Psoriasis vulgaris	Total
Number of patients		50	34	26	39	24	173
Age (years),mean ± SD (range)		28.64 ± 12.86 (6–57)	28.53 ± 8.06 (10–48)	47.46 ± 12.33 (28–80)	43.77 ± 15.12 (10–75)	39.42 ± 14.08 (14–72)	36.35 ± 14.85 (6–80)
Duration of disease (months),mean ± SD (range)		57.75 ± 72.34 (2–276)	45 ± 60.39 (1–240)	54.92 ± 74.43 (1–360)	63.82 ± 79.70 (4–360)	115.92 ± 106.91 (3–360)	
Sex, n (%)	Female	27 (54)	13 (38.2)	17 (65.4)	29 (74.4)	16 (66.7)	102 (59)
	Male	23 (46)	21 (61.8)	9 (34.6)	10 (25.6)	8 (33.3)	71 (41)
Skin phototype,n (%)	I	1 (2)	-	-	1 (2.6)	-	2 (1.2)
	II	23 (46)	11 (32.4)	7 (26.9)	12 (30.8)	8 (33.3)	61 (35.3)
	III	18 (36)	15 (44.1)	14 (53.8)	25 (64.1)	15 (62.5)	87 (50.3)
	IV	8 (16)	8 (23.5)	5 (19.2)	1 (2.6)	1 (4.2)	23 (13.3)
Previous treatments,n (%)	No treatment	6 (12)	4 (11.8)	3 (11.5)	3 (7.7)	2 (8.4)	18 (10.5)
	Topical	39 (78)	30 (88.2)	23 (88.5)	27 (69.2)	18 (74.8)	137 (79.2)
	Systemic	-	-	-	9 (23.1)	4 (16.8)	13 (7.5)
	Phototherapy	5 (10)	-	-	-	-	5 (2.8)
Treatment frequency,n (%)	1/week	-	-	-	3 (7.7)	-	3 (1.7)
	2/week	30 (60)	10 (29.4)	9 (34.6)	22 (56.4)	10 (41.7)	81 (46.8)
	3/week	20 (40)	24 (70.6)	17 (65.4)	13 (33.3)	14 (58.3)	88 (50.9)
	4/week	-	-	-	1 (2.6)	-	1 (0.6)

### 3.1. Vitiligo 

From 50 patients with vitiligo, the targeted phototherapy was used as a monotherapy in 25 patients, whereas the remaining 25 patients received at least one concomitant topical therapy (tacrolimus, corticosteroids, or antioxidants). 

### 3.2. Alopecia areata

Of the 34 patients with AA included in the study, the targeted phototherapy was used as a monotherapy in all patients, except for 1 patient who received concomitant topical tacrolimus treatment. 

### 3.3. Lichen simplex chronicus

**Figure 1:**
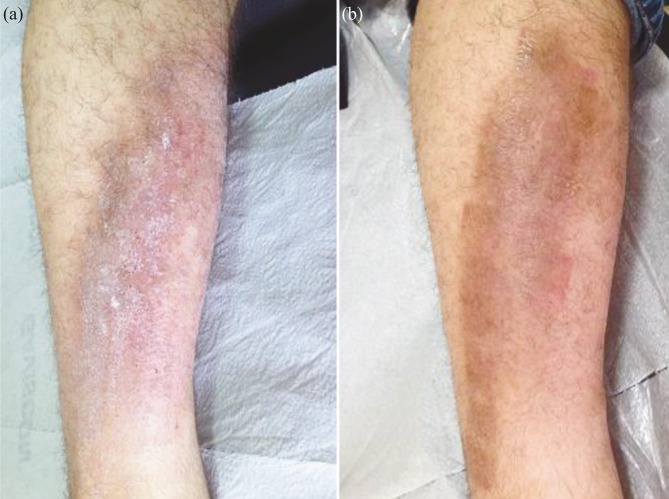
Patient with lichen simplex chronicus (a), showing an excellent response with residual hyperpigmentation (b) at the end of 32 sessions of treatment.

Among the 26 patients with LSC, the targeted phototherapy was used as a monotherapy in all patients, except for 8 patients who were also treated with topical tacrolimus, corticosteroids, and antihistamines (Figure 1). 

### 3.4. Palmoplantar psoriasis/eczema (PPP)

A total of 39 patients with palmoplantar psoriasis/eczema were included in the analysis. The targeted phototherapy was used as a monotherapy in 19 patients, whereas it was combined with topical agents in 17 patients and acitretin in 3 patients.

### 3.5. Psoriasis vulgaris 

**Figure 2:**
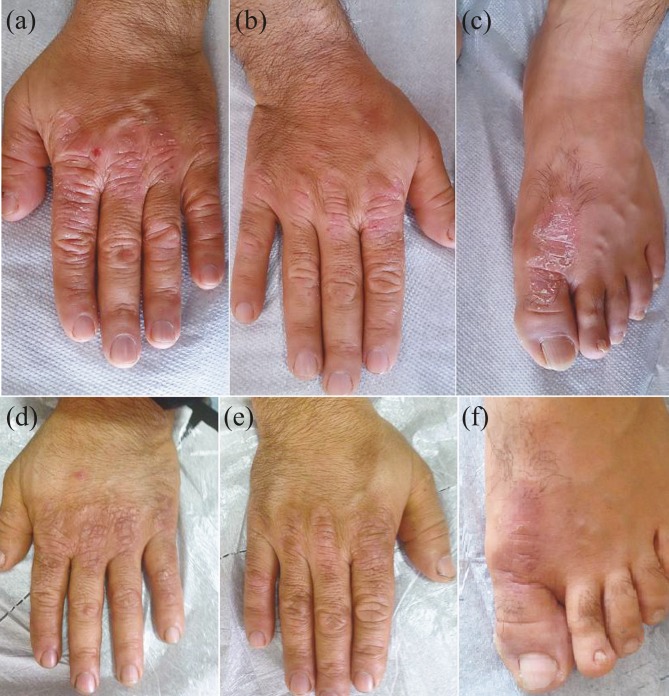
Patient with psoriasis displaying moderate improvement of hand and foot lesions, respectively (a–f), following 25 sessions of treatment.

Of the 24 patients with plaque-type psoriasis vulgaris, the targeted phototherapy was used as a monotherapy in 8 patients, whereas it was combined with topical agents in 15 patients and acitretin in 1 patient (Figure 2).

**Table 2:** Phototherapy parameters, adverse effects, and treatment outcomes in treated patients.

Characteristics		Vitiligo	Alopecia areata	Lichen simplex chronicus	Palmoplantar psoriasis/eczema	Psoriasis vulgaris
Number of patients		50	34	26	39	24
Minimal erythema dose (mJ/cm2),mean ± SD (range)		372.36 ± 116.81 (220–700)	417.32 ± 161.26(130–660)	387.5 ± 132.23(165–660)	331.92 ± 119.41(165–700)	403.96 ± 148.6(145–700)
Initial dose (mJ/cm2),mean ± SD (range)		138.38 ± 63.54 (68–350)	207.12 ± 80.38 (70–330)	170.69 ± 47.04 (80–250)	161.51 ± 62.13 (70–350)	194.42 ± 76.60 (72–350)
Cumulative dose (mJ/cm2),mean ± SD (range)		17589 ± 21706 (90–93730)	18168 ± 16017 (710–71574)	23772 ± 28010 (365–117644)	17070 ± 20202(70–89870)	18328 ± 15998 (204–50391)
Maximum dose (mJ/cm2),mean ± SD (range)		905 ± 609 (90–2510)	1169 ± 574 (300–2665)	1028 ± 647 (144–2988)	1074 ± 706(70–2748)	1201 ± 616(120–2146)
Total number of sessions, mean ± SD (range)		25.40 ± 19.03 (1–82)	23.76 ± 13.60 (2–54)	29.08 ± 24.24 (2–91)	19.54 ± 18.66(1–104)	20.25 ± 11.75 (2–45)
Adverse effects,n (%)	None	22 (44)	9 (26.5)	18 (69.2)	32 (82.1)	12 (50)
	Mild erythema	16 (32)	18 (52.9)	6 (23.1)	7 (17.9)	10 (41.6)
	Moderate erythema	8 (16)	6 (17.6)	2 (7.7)	-	1 (4.2)
	Severe erythema/bullae	2 (4)	1 (2.9)	-	-	1 (4.2)
	Hyperpigmentation	2 (4)	-	1 (3.8)	-	-
Number of patients		46	32	23	33	23
Treatment outcome in all patients assessed, n (%)	No response	28 (61)	9 (28.1)	4 (17.5)	7 (21.2)	2 (8.7)
	Mild	7 (15.2)	10 (31.3)	2 (8.7)	7 (21.2)	8 (34.8)
	Moderate	6 (13)	4 (12.5)	7 (30.4)	4 (12.1)	4 (17.4)
	Significant	2 (4.3)	-	3 (13)	11 (33.4)	7 (30.4)
	Complete	3 (6.5)	9 (28.1)	7 (30.4)	4 (12.1)	2 (8.7)
Number of patients		30	17	13	14	5
Treatment outcome in patients whose treatment was stopped by physician, n (%)	No response	17 (56.7)	4 (23.5)	1 (7.7)	2 (14.3)	-
	Mild	6 (20)	3 (17.6)	1 (7.7)	1 (7.1)	-
	Moderate	3 (10)	1 (5.9)	3 (23.1)	1 (7.1)	-
	Significant	1 (3.3)	-	1 (7.7)	4 (42.9)	3 (60)
	Complete	3 (10)	9 (52.9)	7 (53.8)	4 (28.6)	2 (40)
Number of patients				21	29	19
Visual analog scale scores, mean ± SD	Pretreatment			7.84 ± 2.26	4.55 ± 3.22	5.79 ± 3.66
	Posttreatment			1.96 ± 2.4	1.79 ± 2.96	2.79 ± 3.08

For all patient groups, the demographic and treatment characteristics are shown in Table 1. The phototherapy parameters, treatment outcomes, and adverse effects are summarized in Table 2.

## 4. Discussion

In this retrospective study, the safety and efficacy of targeted NB-UVB therapy was evaluated in patients with vitiligo, AA, LSC, palmoplantar psoriasis/eczema, or psoriasis vulgaris who were treated at our phototherapy unit between 2014 and 2016. Only a limited number of previous studies have addressed the safety and efficacy of targeted UVB treatment for the aforementioned dermatological disorders. 

Phototherapy is commonly used in the treatment of vitiligo, particularly in head and neck lesions recalcitrant to topical therapies. In the literature, the response rates with conventional phototherapy modalities have been reported to be 78%–100% with PUVA and 41%–100% with NB-UVB. Monochromatic excimer laser (MEL), a targeted phototherapy modality, has been reported to induce ≥75% repigmentation in 16%–52% of patients (7). Among the 46 patients with vitiligo, in whom the treatment response was assessed, a moderate or better response was observed in 11 (23.9%) of them. Of the patients in whom the treatment course was finalized by a physician, a moderate or better response was obtained in 7 (23.3%). In a study that investigated the efficacy of NB-UVB in vitiligo, Menchini et al. treated 734 patients with vitiligo and reported >75% improvement in 70% of patients (8). In a study by Majid and Imran, an excellent response was obtained in approximately 63% of patients who were treated once or twice weekly (9). Likewise, Lotti et al. reported an improvement of ≥75% in 72% of 100 patients (10). In another study, a repigmentation rate of 50%–100% was observed in 31 of 40 patients (11). In contrast to the high response rates reported in these studies, Klahan and Asawanonda reported an improvement of ≥50% in only 15% of 15 patients, which was similar to the results of the present study (12). Another study by Asawanonda et al. reported 26%–50% pigmentation in 29 lesions of 6 patients (13). The low response rates observed in our study can be explained by the following: i) the response rates in different areas of the body could not be evaluated separately due to the retrospective nature of the study, and thus this approach might have underestimated the treatment outcomes; ii) a high number of patients were lost to follow-up; iii) a high number of patients attended treatment irregularly; and iv) the mean number of 25 sessions might have been inadequate to induce a moderate or better response, and thus extension of the treatment period in future studies might provide better results. Consistent with previous studies, no adverse events occurred in 22 (44%) patients with vitiligo, whereas the most common adverse event was mild erythema, which was observed in 16 (32%) patients. These findings demonstrate that targeted NB-UVB therapy is a safe treatment in terms of acute adverse effects.

Although uncontrolled studies using conventional phototherapy modalities have reported an improvement rate of 15%–70%, high recurrence rates, lack of randomized controlled trials, and an increased risk of malignancy with the use of PUVA treatment limit the use of conventional phototherapy for AA. Targeted excimer laser therapy is an important treatment alternative with response rates up to 41.5%, particularly in limited, patchy AA (3,14,15). To the best of our knowledge, the present study is the first to investigate the safety and efficacy of targeted NB-UVB therapy for AA. Bayramgürler et al. used cabin-type phototherapy with NB-UVB for AA, and an excellent response was obtained in 6 of 25 patients at the end of 46 treatment sessions. However, because intramuscular corticosteroid therapy was used concomitantly in 4 of 6 patients, the authors concluded that NB-UVB was ineffective for use as a monotherapy for AA (16). In our study, a moderate or better improvement was obtained in 13 of 32 patients (40.6%) after a mean of 23.76 treatment sessions. The response rates were even higher when the patients lost to follow-up were excluded from the evaluation, with 9 of 17 (52.9%) patients showing a complete response. The higher response rates observed in the present study may be explained by the ability of the targeted NB-UVB device to deliver higher doses of NB-UVB. The response rates obtained in the present study are comparable to those of studies using PUVA and targeted MEL; this suggests that targeted NB-UVB monotherapy is an effective treatment option for AA. The present study has two limitations: first, there was no control group due the retrospective design of the study; second, spontaneous remission may occur in AA. Despite these limitations, it can be said that the response rates observed in the present study can be attributed to NB-UVB therapy as a majority of the patients had long-standing disease that was recalcitrant to topical and/or intralesional treatments. In agreement with previous studies, severe side effects and discontinuation of treatment due to side effects occurred in only one patient (2.9%), which supports the safety of phototherapy modalities in patients with AA. 

The efficacy of conventional phototherapy modalities in the treatment of pruritic conditions such as prurigo nodularis and uremic pruritus has been evidenced in many studies (17,18). Regarding the efficacy of phototherapy in the treatment of LSC, only a single study was found in the literature. This study involved the use of targeted 308-nm MEL therapy to treat 6 patients with LSC. The results showed a complete response in one patient and a partial response in the others (19). In another case, targeted NB-UVB therapy was successfully used in a patient with vulvar LSC (20). To the best of our knowledge, the present study is not only the first to evaluate targeted NB-UVB therapy for LSC, but it also has the highest number of patients in comparison to other studies investigating the efficacy of phototherapy for LSC. In the present study, a mean of 29 treatment sessions provided a moderate or better response in 17 of 23 patients (73.8%) who were previously unresponsive to topical treatments. After excluding 10 patients who were lost to follow-up from the analysis, among the remaining 13 patients, 11 (84.6%) showed a moderate or better response and 7 (53.8%) showed a complete response. In addition to objective evidence of efficacy, the posttreatment decrease in VAS scores confirms the subjective efficacy of targeted NB-UVB therapy for LSC resistant to topical measures. Among the responders, the mean number of sessions required to obtain the first and excellent response were 10 and 19, respectively (data not shown). These findings indicate that targeted NB-UVB can provide a relatively rapid response that may improve patients’ adherence to treatment. In addition, targeted NB-UVB therapy has demonstrated a good safety profile in our study, with no patients experiencing any severe side effects or leaving treatment due to side effects.

The successful use of phototherapy modalities, including PUVA, UVA1, and MEL, for the treatment of palmoplantar psoriasis and pustulosis has been demonstrated in numerous studies (21–24). The present study is the third study in the literature to investigate the use of targeted NB-UVB therapy for PPP. Previously, Sezer et al. used a right-left comparative study to compare the efficacy of PUVA and local NB-UVB in 25 patients; this study reported an improvement rate of 85% and 61% with PUVA and local NB-UVB, respectively, after 27 treatment sessions (25). Kawada et al. reported an improvement rate of 61.4% in 15 patients with palmoplantar psoriasis/pustulosis after a mean of 13.7 treatment sessions (26). In accordance with the literature, in the present study, a moderate or better response was observed in 19 (57.5%) of 33 patients after a mean of 19.54 treatment sessions. When the patients lost to follow-up were excluded, a complete and moderate or better response was observed in 4 (28.6%) and 9 (68.6%) patients, respectively. The higher rates of response observed in the present study are most likely due to a combination of targeted NB-UVB with topical treatments and acitretin in 17 (43.7%) and 3 (7.7%) patients, respectively. However, because a moderate or better response was obtained in 10 (62.6%) of 16 patients who received targeted NB-UVB as a monotherapy, it is conceivable that targeted NB-UVB therapy could be effectively used in patients with PPP as either a monotherapy or in combination with topical treatments. Considering the lower penetration of NB-UVB compared to PUVA (1), future studies investigating the efficacy of combined treatment with systemic/topical treatments and NB-UVB are required, particularly in the treatment of hyperkeratotic lesions. The decrease in the VAS scores following treatment confirms that targeted NB-UVB therapy is also effective subjectively. In agreement with the literature, 32 (82.1%) of patients in the present study did not experience any side effects, and the remaining 7 (17.9%) demonstrated only mild erythema, a result that implies the safety of targeted NB-UVB therapy.

According to treatment guidelines, cabin-type phototherapy with PUVA and NB-UVB phototherapy are listed among the first-line therapies for moderate to severe psoriasis (21,27). Regarding targeted phototherapy modalities, numerous studies have demonstrated the efficacy of 308-nm MEL therapy (4,6). However, there have been limited studies on the efficacy of targeted UVB therapy for plaque-type psoriasis, and, in most of these, broad-band UVB (BB-UVB) has been administered. In a study by Toll et al., 15 patients were treated with BB-UVB and a complete response was observed in 3 patients and an almost complete response in 5 patients (28). Lapidoth et al. reported a 73% decrease in the psoriasis severity index in 28 patients with psoriasis at the end of 18 sessions of BB-UVB treatment (29). Kemeny et al. compared the efficacy of high- and low-dose BB-UVB in 20 patients and observed a 93% and 84% improvement, respectively (30). In addition to studies involving BB-UVB, several studies have investigated the efficacy of targeted NB-UVB therapy for plaque-type psoriasis (31–33). For instance, Amornpinyokeit and Asawanonda compared the efficacy of targeted NB-UVB monotherapy and the combination of targeted NB-UVB therapy and 8-methoxypsoralen cream in 10 patients with psoriasis and found the combination to be more effective (31). The present study had the highest number of patients in the literature, with 24 patients with psoriasis having been treated. After a mean of 20.25 treatment sessions, a moderate or better response was observed in 13 patients (56.5%). When patients who had any concomitant treatment were excluded from the analysis, a moderate or better improvement was observed in 4 (50%) out of 8 patients. Although the response rates observed in our study are lower than those reported in BB-UVB studies, they are higher than those with only DB-UVB treatment. The VAS scores for pruritus decreased from 5.79 to 2.79 following treatment. The results from the present study suggest that targeted NB-UVB therapy is an effective and safe alternative for treating localized, recalcitrant psoriatic lesions, either as monotherapy or combined with topical treatments.

The present study has some limitations, mostly arising from its retrospective design. First, the absence of untreated control lesions is a limiting factor in the evaluation of treatment response, particularly in AA and vitiligo, which are likely to resolve spontaneously. However, the fact that the patients included in the study had a long duration of disease and stable lesions reduces the likelihood of spontaneous remission. Furthermore, although rare, the occurrence of device breakdowns during the treatment course might have negatively influenced the treatment continuity, patients’ adherence to treatment, and thus the treatment outcomes. An additional limitation of the current study is the determination of MED on healthy skin in all patients. Determination of MED in vitiligous skin might have decreased the rate of adverse effects and increased the patient adherence. Another limitation of our study is that in patients who were treated for more than one lesion, the treatment response of lesions located in different anatomical regions was not compared. This might have resulted in lower than expected response to treatment, particularly in patients with vitiligo. In future studies, it would be of interest to compare the efficacy of targeted NB-UVB therapy in various anatomical sites and to evaluate the treatment response of individual lesions rather than a global assessment. The lack of a follow-up period for patients included in our study is another limiting aspect of our research. Finally, the response rates between the patients who received targeted NB-UVB therapy, as either a monotherapy or concomitant with topical and/or systemic agents, were not compared because of the small sample size. A prospective study is planned to compare the efficacy of various treatment combinations in different conditions. The larger sample size of the present study in comparison to previous studies and the use of standardized treatment protocols are two main advantages. Another strength is the fact that targeted NB-UVB therapy was used for the first time for AA and LSC.

In conclusion, our findings demonstrate the safety and efficacy of targeted NB-UVB therapy for vitiligo, AA, LSC, palmoplantar psoriasis, and plaque-type psoriasis. Results suggest that NB-UVB is a safe and highly effective treatment option, particularly in AA, LSC, and palmoplantar and plaque-type psoriasis recalcitrant to topical treatments. In the future, prospective, randomized, controlled studies investigating the efficacy of different treatment combinations as well as the influence of patient- and disease-related factors on response rates are warranted.
